# Evaluation of lung function in patients submitted to total laryngectomy^☆^^☆☆^

**DOI:** 10.1016/j.bjorl.2018.05.008

**Published:** 2018-06-29

**Authors:** Mario A. Castro, Rogério A. Dedivitis, João M. Salge, Leandro L. Matos, Claudio R. Cernea

**Affiliations:** a Universidade de São Paulo (USP), Faculdade de Medicina, Departamento de Cirurgia de Cabeça e Pescoço, São Paulo, SP, Brazil; b Universidade de São Paulo (USP), Faculdade de Medicina, Instituto do Coração (INCOR), Departamento de Pneumologia, São Paulo, SP, Brazil

**Keywords:** Respiratory function tests, Spirometry, Plethysmography, Laryngectomy, Squamous cell, Testes de função respiratória, Espirometria, Pletismografia, Laringectomia, Célula escamosa

## Abstract

**Introduction:**

The post-laryngectomy state is characterized by several alterations in lung function. A reliable estimation of lung function can be very useful in laryngectomees to prevent postoperative complications and to evaluate the results of the treatment.

**Objective:**

Characterize the presence of respiratory functional disorders and the functional pattern of laryngectomees through the use of an extratracheal device.

**Methods:**

This transversal study included 50 patients submitted to total laryngectomy at least 6 months prior to this investigation, as the treatment of choice for laryngeal cancer.

**Results:**

56% percent of the participants had altered breathing pattern, distributed as follows: 14 with obstructive pattern with no air trapping, 11 with obstructive pattern with air trapping and only 3 with restrictive pattern. On average, the diffusion decreased (74.3%) and airway resistance increased (121.7%) when compared to the expected average values for the Brazilian individuals.

**Conclusion:**

Most patients submitted to total laryngectomy present altered lung function, usually the obstructive type, frequently associated to a history of smoking.

## Resumo

**Introdução:**

A condição pós-laringectomia é caracterizada por várias alterações na função pulmonar. Uma estimativa confiável da função pulmonar pode ser muito útil em pacientes laringectomizados para prevenir complicações após as intervenções cirúrgicas e avaliar os resultados do tratamento.

**Objetivo:**

Caracterizar a presença de distúrbios funcionais respiratórios e o padrão funcional de pacientes laringectomizados através do uso de um dispositivo extratraqueal.

**Método:**

Estudo transversal que incluiu 50 pacientes submetidos à laringectomia total pelo menos seis meses antes desta investigação, como tratamento de escolha para o câncer de laringe.

**Resultados:**

Dos participantes, 56% apresentavam padrão respiratório alterado, assim distribuídos: 14 com padrão obstrutivo sem aprisionamento aéreo, 11 com padrão obstrutivo e aprisionamento aéreo e apenas três com padrão restritivo. Em média, verificou-se que a difusão encontrava-se diminuída (74,3%) e a resistência das vias aéreas aumentada (121,7%) em relação aos resultados esperados em brasileiros.

**Conclusão:**

A maioria dos pacientes submetidos à laringectomia total apresenta função pulmonar alterada, do tipo obstrutiva, quase sempre associada a história de tabagismo.

## Introduction

Many patients with larynx cancer submitted to surgical treatment present a history of smoking. In these patients, pulmonary complications can lead to death in the postoperative period. Around 81% of them suffer from chronic obstructive pulmonary disease (COPD).^1^, ^2^

The evaluation of respiratory function in laryngectomized patients has been the subject of study since Heyden's paper of 1950.^3^ Due to technical difficulties for the performance of this evaluation, the literature on this subject is controversial.

Due to a history of smoking before laryngectomy as well as the non-physiological conditions of the airways, the post-laryngectomy state presents several alterations in lung function, reflected in the sum of ventilatory changes. The air inhaled through the tracheostoma after total laryngectomy does not go through the natural conditioning of the upper respiratory tract; hence the filtration of solid particles transmitted by both air and aerosols is reduced. Furthermore, the inhaled air is submitted neither to humidification nor heating. In comparison with respiration through the upper respiratory tract, the tracheotomized patient has an aerodynamic reduction in the resistance to the airflow during inspiration and expiration and this may cause a negative effect in the peripheral ventilation of the lung.^4^, ^5^ One of the most important prognostic factors in the survival of laryngectomized patients is the progressive deterioration of their lung function.^6^, ^7^

A reliable estimation of lung function can be very useful in laryngectomees to prevent complications occurring after in surgical interventions, to evaluate the results of the treatment and even for preventive purposes.^8^

A tracheal cannula with a cuff connected to the spirometer is normally used to evaluate lung function in these patients.^1^, ^6^, ^9^ However, the use of a cannula is not the ideal option. First, it is an uncomfortable experience for the patient and leads to coughing.^5^ Furthermore, due to a decrease in the real diameter of the trachea, the results of forced expiration and inspiration tests are not precise. The use of tracheal masks manually placed over the tracheotoma has already been reported for the same purpose; however, they permit air leakage.^2^

Few of the papers found in the literature have evaluated lung function in laryngectomized patients with extratracheal devices making use of the reproducible methodology.^10^

The aim of this study is to characterize the presence of respiratory functional disorders and the laryngectomees functional pattern through the use of an extratracheal device.

## Methods

This research was approved by the Institutional Review Board, under number 075/14, on May 12th, 2014.

This transversal study included 56 patients who had undergone total laryngectomy at least 6 months prior to this investigation, as the treatment of choice for laryngeal cancer. The patients were enrolled between March and June, 2014.

Exclusion factors were: acute respiratory disease in the previous 30 days; the absence of clinical conditions at the time the tests were carried out; and the inability to carry out any test in the study.

Demographic data were obtained, including a history of smoking prior to surgery and occasional respiratory difficulties (Dyspnea Scale – Medical Research Council),^11^ through patient recollection and medical records.

Tests were carried out with subjects in the sitting position, connected to the spirometer by an adhesive extratracheal device with a silicone adaptor for hands-free type valves (Provox®, Atos Medical, Horby, Sweden)^10^ since participants were laryngectomized, thus making it impossible to perform a conventional lung function test with a nozzle.

The apparatus used for the lung function tests and pletysmography was Elite DX (Medical Graphics Corporation®, Saint Paul, MN, USA). The performance of the maneuvers as well as the selection of results followed the criteria established in the Guidelines for Lung Function Tests.^12^, ^13^, ^14^

Inspiratory and expiratory loops of the X-flow curve were obtained through maneuvers of Forced expiratory Vital Capacity (FVC) in which the subject performs a forced expiration, with no hesitation, starting from the Total Lung Capacity (TLC) up to the Residual Volume (RV). Selected values for FEV_1_ (Forced Expiratory Volume in 1 second)/FVC were expressed as percentage of the normal values calculated according to the European Respiratory Society recommendation.^15^ In addition, the maneuver of slow vital capacity was carried out, in which the subject performs a complete expiration starting from the maximum pulmonary insufflations, but without using maximum expiratory effort. The results of this maneuver were used in the composition of the calculation of pulmonary volumes.

The tests were interpreted by a specialist in spirometry and classified as: normal, obstructive breathing disorders with no arrest, obstructive breathing disorders with arrest and restrictive breathing disorders.

Thoracic Gas Volume (TGV) was measured using the whole body pletysmography technique for obtaining pulmonary volumes.^16^, ^17^ RV and TLC were calculated from TGV. The obtained results were expressed as percentage of the normal calculated values and later adjusted to the 2008 table of expected values for Brazilians according to their gender.

For the characterization of the subjects’ sample regarding lung parenchyma impairment, the measurement of lung diffusion was made through the classic technique of single breath using carbon monoxide (CO),^16^ associated with the most recently incorporated recommendations.^12^ The maneuver was repeated at least twice up to a maximum of five times. Results were expressed as a percentage of expected values for Brazilians according to their gender. The maneuver for measuring airway resistance was performed using the whole body pletysmography technique.^18^ The conductibility of the airways was calculated (Gaw = 1 raw) using the raw measure, as well as the specific conductibility of the airways obtained through its correction for the pulmonary volume in which the measurement was carried out (sGaw = Gaw/TGV). Raw and sGaw values were expressed as cm H_2_O/L/s and 1 cm H_2_O/s, respectively.

The distribution of frequencies was used to describe the categorical variables (number of cases and percentage) and the measures of central tendency (mean and median) and variability (minimum, maximum and standard deviation) for the continuous or numerical variables.

## Results

Of the 56 participants of this study, 6 were excluded; 3 for not being able to perform the requested maneuvers during the tests and the others due to their diminished cognitive condition, which impeded understanding of instructions.

Table 1 describes the demographic characteristics and history of smoking (before surgery) of the evaluated laryngectomized subjects. Average age was 64, with 88% of subjects being men; 58% of participants smoked more than 40 cigarettes a day.

**Table 1:** Demographic and smoking characteristics of the laryngectomized.

Variable	Category/measures	Freq. (%)/measures
Age (years)	Variation	41–87
	Median	64.5
	Mean (standard deviation)	64.2 (10.3)

Race	Orientals	2 (4)
	Afrodescendents	12 (24)
	Caucasians	36 (72)

Body mass index (kg/m^2^)	Variation	15.7–32.6
	Median	24.2
	Mean (standard deviation)	24.4 (3.8)

Gender	Female	6 (12.0)
	Male	44 (88.0)

Smoking	Non smoker	6 (12.0)
	Less than 10 cigarettes/day	4 (8.0)
	10 to 20 cigarettes/day	7 (14.0)
	21 to 40 cigarettes/day	4 (8.0)
	More than 40 cigarettes/day	29 (58.0)

Regarding the Dyspnea Scale (Medical Research Council),^11^ participants were stratified according to Table 2. Of the 56 participants, 28 presented dyspnea only with strenuous exercise and 19 when hurrying on the level or walking up a slight hill.

**Table 2:** Estratification of the participants regarding dyspnea scale.

Dyspnea scale	Freq. (%)
Breathless with strenous exercise	28 (56)
Short of breath when hurrying on the level or walking up a slight hill	19 (38)
Walks slower than people of the same age on the level or stops for breath while walking at own pace on the level	2 (4)
Stops for breath after walking a 100 yards	1 (2)

Respiratory functional measures are described in Table 3, which demonstrates an average percentage of the FVC and FEV1 below that expected for Brazilians. On average, diffusion was found to be reduced and airway resistance increased.

**Table 3:** Respiratory functional measures.

Variable	Category/measures	Absolut values	Percentage values
	**Spirometry**		
FVC = Forced expiratory vital capacity	Variation	1.7–4.9	521–1096
	Median	3.4	841
	Mean (standard deviation)	3.3 (07)	833 (126)

FEV_1_ – Forced expiratory volume in 1 second	Variation	1.1–3.5	43.4–106.7
	Median	2.5	77.5
	Mean (standard deviation)	2.4 (0.6)	75.8 (15.1)

FEV_1_/FVC	Variation	49–90	66,6–109,5
	Median	71.5	91.9
	Mean (standard deviation)	71.4 (8.8)	91.1 (11.1)

	**Lung Volumes**		
RV = Residual volume	Variation	1.3–4.0	63.3–240.1
	Median	2.4	112.3
	Mean (standard deviation)	2.5 (0.6)	122 (32.3)
TLC = Total lung capacity	Variation	3.4–8.1	71.39–126.4
	Median	6.0	98.3
	Mean (standard deviation)	6.0 (1.0)	99.2 (12.3)

	**Diffusion**		
DLCOunc = Diffusing capacity of the lung for carbon monoxide	Variation	9.8–34.3	42.1–124.9
	Median	20.6	20.6
	Mean (standard deviation)	21.0 (6.5)	21.0 (6.5)

	**Airway resistance**		
Sgaw = Specific airway conductance	Variation	0.1–0.5	50–185
	Median	0.2	105
	Mean (standard deviation)	0.2 (0.1)	121.7 (52.8)

Values in percentages of those expected for Brazilians.

The results obtained after interpretation and classification of breathing patterns are described in Table 4. 56% percent of the participants were found with altered breathing pattern, distributed as follows: 14 with obstructive pattern with no air trapping, 11 with obstructive pattern with air trapping and only 3 with restrictive pattern.

**Table 4:** Frequency and percentage of breathing patterns.

Interpretation patterns	Freq. (%)
Normal	22 (44)
Obstructive pattern with no air trapping	14 (28)
Obstructive pattern with air trapping	11 (22)
Restrictive	3 (6)

## Discussion

After the probability of dying of a second leading cancer, pulmonary diseases are the second cause of mortality in laryngectomized patients and this could suggest the carrying out of spirometry in these patients.^7^ However, little attention has been given in the literature to the evaluation of lung function in patients submitted to total laryngectomy, especially due to technical difficulties associated with execution of the test and not to lack of indication.^19^

Several studies agree that many laryngectomees could benefit from drug treatment due to the high percentage of airway obstruction.^2^, ^6^, ^7^ According to the Medical Research Council Dyspnea Scale,^11^ 56% of patients reported fatigue only after strenuous exercise and 38% when hurrying on the level or walking up a slight hill. This finding could be related to quitting smoking after surgery, since the effect of giving up smoking is relatively immediate.^20^

Evaluating the spirometry, several studies have found subnormal values in laryngectomized subjects.^1^, ^6^, ^21^, ^22^ Our results showed an average percentage of CVF and VEF1 below that expected for Brazilians, respectively 83.3% and 75.8%.

These data confirm those collected by Duran et al.,^23^ which found a significant reduction in these indexes.

Data obtained in our investigation agree with other results,^7^ in which an increase in the residual volume and decrease in the FEV1 were found, thus showing an obstructive respiratory condition.

On average, diffusion was found to have decreased (74.3%) and airway resistance increased (121.7%) in our results in relation to expected Brazilian results.

After carrying out lung function tests, 44% of patients were found with normal breathing pattern and 56% altered, distributed among 14 patients with obstructive pattern with no air trapping, 11 with obstructive pattern with air trapping and only 3 with restrictive pattern. Our figures agree with those found by Duran et al.^23^ These authors found that in their series with 30 laryngectomized patients, 47% presented normal lung function, whereas 53% had altered lung function. In the series by Vasquez et al.,^7^ 81% of patients were found with lung function of obstructive breathing pattern. As justified by Ackerstaff et al.,^5^ perhaps the greatest index of respiratory dysfunction found in laryngectomized patients is due to a greater number of possible respiratory infections due to the tracheostoma.

Our results agree with the study performed by Hess et al.,^2^ showing that it might be necessary to reevaluate the role of the lung function test in the monitoring of patients submitted to total laryngectomy.

The use of a standardized extra-tracheal device with reproducible methodology is essential to guarantee the precision and accuracy of lung function tests in patients submitted to total laryngectomy.

## Conclusion

Most patients submitted to total laryngectomy present altered lung function, of the obstructive type, most of the time due to a history of smoking.

## Conflicts of interest

The authors declare no conflicts of interest.
